# Sea-Sickness, a Remedy in Certain Cases of Jaundice

**Published:** 1844-10-19

**Authors:** H. Percy

**Affiliations:** Worthington


SEA-SICKNESS, A REMEDY IN CERTAIN CASES OF JAUNDICE.

BY H. PERCY, ESQ., WORTHINGTON.

Emma Cox, aged twenty-eight, cook in a gentleman's family, of a full habit, had been suffering from jaundice between three and four months; previous to this attack had always enjoyed good health. When I first saw her the skin was deeply dyed, bowels obstinately constipated with pain in the epigastric region, recurring at intervals, but not of a very severe character; the motions had not the least tinge of bile. After attending on her a considerable time without at all relieving her, I one day, when the sea was rough, recommended her sailing in a boat for three or four hours; she at once followed the advice, and, to use her own words, was horribly sick. I afterwards administered a dose of brisk aperient every four hours; within twenty-four hours she discharged, by stool, several small gall-stones, and a large quantity of inspissated bile. The complexion rapidly became clear, and she soon got perfectly well, an occasional aperient being the only medicine used. I have not as yet had an opportunity of trying this remedy a second time, but where there is only mechanical obstruction to the passage of the bile, I think the relaxing effects of sea-sickness must tend to accelerate the stone through the duct.

In the above case the patient was cured at once by it, and were it not so immediately beneficial (as will most probably generally be the case) it might be persevered in, with intervals of a few days, with a reasonable prospect of success.—*Ibid.*

